# On Healthy and Diseased Bone, According to the Investigations of Frerichs, Marchand, Nasse, Ragsky, and Simon

**Published:** 1844-01-01

**Authors:** 


### On Healthy and Diseased Bone; according to the Investigations of *Frerichs, Marchand, Nasse, Ragsky*, and *Simon*. (Beiträge zur Physiologischen und Pathologischen Chemie und Mikroscopie, v. Dr. *Fr. Simon* Band 1. Lief. 2. Berlin, 1843.)

The examination of the bones extends as well to their combustible as to their incombustible constituents. The mode in which the latter are deposited in the structure of the former, must necessarily be taken into the account; for this reason, a microscopical must always precede the chemical examination of the bones, more especially when we have to deal with pathological changes. The organic material of the bone must be examined, as to whether it yields jelly (leim) or not by boiling; whether this jelly is quick or slow in forming, whether it is gluten or chondrin; or whether it manifests any other important property on the application of re-agents. This watery decoction may also be examined with advantage to ascertain the quantity of alkaline sulphates or phosphates, or even of earthy phosphates, by boiling for a considerable time in nitric acid, filtering, and then testing with ammonia for earthy phosphates, with a barytic salt for sulphuric and phosphoric acid. The other preparatory steps we shall not detail, as they are only interesting to the mere chemist.

### On Healthy and Diseased Bone.

On the Subject of Healthy Bone, several experiments have been instituted by Frerichs, (Annal. d. Chemie u. Pharmacie, Septbr. 1842) and by Marchand (Journal f. Praktische Chemie, Oktbr. 1842.)

In order to ascertain whether the composition of bone is essentially invariable, Marchand experimented on the thigh-bone of a man thirty years of age, after first divesting it of the periosteum and fat. The result of the analysis coincides so closely with the results obtained some years ago by Berzelius, that the invariable composition of the compact bones at least seems extremely probable. These results were as follow:—

| | Berzelius. | Marchand. |
|---|---|---|
| Cartilage perfectly soluble in water . . | 32,17 .. | 27,23 |
| „ insoluble in hydrochloric acid . | — .. | 5,02 |
| „ soluble „ „ | — .. | 1,01 |
| Vessels . . . . . . . . . . . . | 1,13 .. | — |

| | Berzelius. | | Marchand. |
|---|---|---|---|
| Basic phosphate of lime with a little fluoride of calcium | 53,04 | .. | — |
| Basic phosphate of lime | — | .. | 52,26 |
| Fluoride of calcium | — | .. | 1,00 |
| Carbonate of lime | 10,30 | .. | 10,21 |
| Phosphate of magnesia | 1,16 | .. | 1,05 |
| Soda, with a little muriate of soda | 1,20 | .. | — |
| Soda | — | .. | 0,92 |
| Chloride of sodium | — | .. | 0,25 |
| Oxide of iron, oxide of manganese. Loss | — | .. | 1,05 |
| | 100,00 | | 100,00 |

As the results of the examinations hitherto made, with respect to the relative proportion of the organic constituents to the incombustible residue in the bones of various parts of the body of one and the same individual, as well as in the bones of persons of different ages, evince but little correspondence, Frerichs took up the investigation of the subject once more. The bones well-cleansed and divested of periosteum, were comminuted, the fat was removed from them with ether, and they were dried in the oil-bath at a temperature of from 130° to 140°, as long as any loss of weight manifested itself; then a determinate quantity was calcined in a platinum crucible, and the carbonic acid, which may probably have escaped by the heat, was compensated with carbonate of ammonia, and the quantity was again weighed.

The experimenter employed bones which had been previously subjected to a maceration by means of lime; in this way those bones were avoided, in which a portion of the cartilage was destroyed in consequence of this treatment, as well as those which may have inclosed in their cavities any part of the lime employed in macerating; the bones of the adult belonged to one and the same individual. The cortical and medullary substance were taken equally for the purpose of analysis.

| | Inorg. Consts. | | Org. Consts. |
|---|---|---|---|
| Parietal bone of an adult | 68,5 | .. | 31,5 |
| „ „ a child three years old | 66,3 | .. | 33,7 |
| Petrous portion of temporal bone of an adult | 70,2 | .. | 29,8 |
| Inferior maxilla of an adult | 68,0 | .. | 32,0 |
| „ „ a child three years old | 62,8 | .. | 37,2 |
| Sternum of an adult | 64,7 | .. | 35,3 |
| Rib | 65,3 | .. | 34,7 |
| Humerus of an adult | 68,3 | .. | 31,7 |
| Humerus and ulna of an eight months' fœtus | 63,2 | .. | 36,8 |
| Radius of an adult | 66,3 | .. | 33,7 |
| Radius of a boy ten years old | 65,5 | .. | 34,5 |
| Tibia of an adult | 66,2 | .. | 33,8 |
| Fibula of an adult | 66,5 | .. | 33,5 |
| Carious excrescence of another fibula | 61,2 | .. | 38,8 |
| Metatarsal bone of an adult | 65,9 | .. | 34,1 |
| Patella of an adult | 63,7 | .. | 36,3 |
| Body of a lumbar vertebra of an adult | 60,5 | .. | 39,5 |

From these investigations the author infers—

1st. That the quantity of lime contained in the bones of different parts of the body of one and the same individual is different.

2nd. That the fixed salts are so much the less, in proportion as the medullary canals and cavities increase, and accordingly less in the spongy bones than in the compact bones; however the experimenter seeks for the greater quantity of organic matter contained in the spongy bones, not in a greater quantity of cartilage, but in that of the membrane and vessels, which line the medullary cavities and canals.

3rd. That the inorganic constituents increase with age. Schreyer found in the bones of the child 48,48, of fixed salts, in those of the adult 74,84, in those of the old man, 84,10; so considerable an increase of inorganic matter the author could not discover in the adult, as appears from the preceding series of experiments; for the difference here amounts to only from two to five per cent.; the proportion of the inorganic substance in the humerus and ulna of an eight months' fœtus where this amounts to 63,2 per cent., and that in the humerus of an adult, where it amounts to 68,3 per cent., is particularly striking.

4th. That the quantity of inorganic substance in the bones is greater than is stated by Rees and some others, probably because in their investigations the water so obstinately retained by the bones had not been completely removed.

The author instituted an experiment in order to ascertain after what manner the carbonate and phosphate of lime are distributed in the bones, or deposited one beside the other, whether the one combination is found exclusively in the osseous corpuscles, the other in the structureless intermediate substance. From a thin lamina of bone in which the osseous corpuscles, and their radiating ramifications were plain to be seen, the cartilage was carefully extracted with a weak alkaline ley, the lamina was cleansed, and moistened with a solution of nitrate of silver. Beneath the microscope the substance was now observed to present everywhere a uniformly yellow colour, a proof that the carbonate and phosphate of lime were equally distributed quite through. In order to determine the question, whether the calcareous salts are chemically combined with the cartilage in the structureless intermediate substance of the bones, or mechanically distributed therein, the experimenter tried such a combination of phosphate of lime with gelatine by mixing a solution of common gelatine and basic phosphate of lime in hydrochloric acid and causing precipitates by ammonia: the precipitate obtained contained 18,5 per cent. of gelatine; when a similar mixture was prepared with a gelatine obtained from bone cartilage, wherein the latter (the gelatine) was applied in excess, from three experiments a mean of 26,5 per cent. of gelatine was obtained. This proportion seems to countenance the idea of a chemical combination of the gelatine with the bone-earth; if one adopt the atomic weight of gelatine as determined by Mulder, a combination of one atom of bone-earth and one atom of gelatine must contain 26,2 per cent. of the latter. In order to discover whether the proportion between the carbonate and phosphate of lime continues the same in the spongy and compact bones, analyses were instituted of the two species of bone of one and the same individuals. The results were—

| | For the Spongy Bone. 1. | 2. | | For the Compact Bone. 1. | 2. |
|---|---|---|---|---|---|
| Organic substance | 38,22 | 37,42 | .. | 31,46 | 30,94 |
| Earthy phosphates | 50,24 | 51,38 | .. | 58,70 | 59,50 |
| Carbonate of lime | 11,70 | 10,89 | .. | 10,08 | 9,46 |
| | 100,16 | 99,69 | .. | 100,28 | 99,90 |

From this it seems to follow that, in the compact bones and in the spongy bones, the relative proportion between the earthy phosphates and carbonate of lime is not entirely the same: in the former, of the sum of both earths the earthy phosphates amounted to 86, and in the other to 82 per cent.

Bones which have been a long time exposed to atmospheric influences, change no doubt, yet not essentially, more especially if they are covered with earth; the beds in which they lie have some influence on their composition, as follows from two comparative analyses made by Marchand with bears' bones, obtained from caves, the one of which lay near the surface, and the other at a considerable depth under ground. The analysis of the deeper-laid bone is marked 2, that of the bone lying at the surface is marked 1; the upper thigh-bone of a fossil deer was also examined by Marchand, the analysis of which is marked 3.

| | 1. | 2. | 3. |
|---|---|---|---|
| Animal substance | 4,20 | 16,24 | 7,25 |
| Earthy phosphates | 62,11 | 56,01 | 54,15 |
| Earthy carbonate | 13,24 | 13,12 | 19,26 |
| Earthy sulphate | 12,25 | 7,14 | 12,24 |
| Fluoride of calcium | 2,12 | 1,96 | 2,08 |
| Phosphate of magnesia | 0,50 | 0,30 | 2,12 |
| Silicic acid | 2,12 | 2,15 | —— |
| Oxide of iron, oxide of manganese, soda, and loss | 3,46 | 3,08 | —— |
| Oxide of iron, oxide of manganese, and loss | —— | —— | 2,90 |
| | 100,00 | 100,00 | 100,00 |

From these investigations it follows that, with the decrease of animal substance, the quantity of the carbonate of lime increases; further, in all the three analyses the quantity of the fluoride of calcium is much more considerable than in the bones of animals of a more recent date.

### ON DISEASED BONE.

Several articles have been published of late years on diseased bones; of the most valuable of these I shall here present an analysis; a series of experiments instituted by Dr. Ragsky, have been published by Rokitansky in the second volume of his pathological anatomy; other experiments have been made by others, more especially by Marchand, Ephraim, Nasse, whilst I myself have directed my attention to the disease called Osteoid.

It will probably not be unacceptable to the generality of my readers, if, in the first instance, I present to them a condensed analysis of the observations made by Rokitanski, who has deserved so well of pathological anatomy, concerning the external characters and internal structure of diseased bone.

1. *Exostosis.*—The compact exostosis appears as a plano-convex knob that has been as it were glued on, which frequently exceeds in hardness and density the bony substance to which it is attached; this, from the very commencement of its formation, possesses equal density, so that the very smallest miliary excrescences, being equally dense as the largest, never appear spongy, and continue their growth in such a manner that the newest strata soon pass into the state of ivory density. They vary in size from that of a smoothened millet-seed to that of a hazel-nut; their surface is generally even, sometimes also uneven, but always smooth, and, as it were, polished; if it grow beyond the ordinary size, it presents sometimes a horny knob, sometimes knobs of a more or less cylindrical form; the colour of these compact exostoses is white, yellowish-white, and whiter than the bone to which it is attached. 2. The spongy exostosis presents a tumour of a cellular texture, filled with marrow, and lined by a compact lamella, as a covering; it is developed sometimes from the compact, sometimes from the spongy substance of the bone; the external covering passes into that of the bone itself. Spongy exostoses are also met, which within the compact external covering exhibit not only the spongy tissue, but also a regular medullary cavity, which communicates with the tubus medullaris. After the spongy exostosis has continued for an indeterminate time in its proper structure, an increase of mass, a sclerosis takes place in it, in various degrees and to various extent; it acquires a compact external covering of considerable density by a stratum of spongy substance, or a regular medullary cavity is inclosed; it becomes also equally compact to a considerable depth in several places, nay even all the way through.

2. *Osteophyte.*—Well-defined lines of distinction cannot be drawn between exostosis and osteophyte; still the latter has so many obvious peculiarities, that in the generality of cases it may be readily distinguished from exostosis. Contrary to exostosis, the osteophyte appears as a bony structure, which in most cases involves extensive portions of a bone, and covers it in various forms. The osteophyte appears velvet-like and villous, when it covers the bone like a ring, or as a stratum from one to two lines thick, which consists of fine fibrils and lamellæ, and thereby puts on the appearance of velvet or of a fine felt. Whilst it is growing thicker, it acquires a smooth external covering perforated by numerous fine pores, and at some depth it acquires an evident lamellated structure. The colour of this osteophyte in the recent state is blue, rose-red, inclining to yellow, a dirty white, or of a colour blending white with a silken or asbestos-like gloss, according to the intensity of the process by which it was formed, according to its duration, and the process of its ossification. The osteophyte presents the splintery-leaved appearance, when it covers the bone in the form of conical excrescences or lamellæ several lines in length, which, beneath a fine, porous, compact external covering, contain a large-celled osseous structure, or even one simple cavity. The warty osteophyte forms wart-like excrescences with a broad or narrow base, consisting of a chalky, white and very brittle substance. (Generally appertaining to the hip-joint, and its arthritic metamorphosis.) The osteophyte in the form of smooth, styloid, knotty prolongations, simple or ramified, pedunculated and round, is hard and of a thick texture. The osteophyte which appears in the form of a bony mass poured on the bone, and solidified as it were at the moment of its flowing, with even and smooth, or uneven and smooth surface, is also compact.

*Diseases of the Bony Texture.*

3. *Osteitis.*—Inflammation of bone may arise from external or internal causes, the latter are more particularly connected with the state of dyscrasis. This form of inflammation has its seat sometimes in the compact, sumetimes in the spongy substance. A very moderate degree of inflammation throws out gelatinous exudation, which passes from a dark red through the yellow red, into a reddish-white, and ultimately into a white colour, which exudation, with respect to its consistence, passes from that of a gelatinous to that of a pliant, flexible cartilage, and of a reddish-white succulent bone; this covers the bone according to its quantity, as a scarcely perceptible white porous growth, or in the form of a very fine felt or velvet, and is connected internally with the bone as with the periosteum. More violent, repeated, or, as it would appear, specific inflammations of bone, give out more copious exudations of the form above-mentioned, whereby the periosteum is increased sometimes into a fibrous callus or enormous thickness, as is occasionally observed on the shin-bone. Beneath the periosteum, the base of the ulceration, there is found a growth or secretion, consisting of curled or straight osseous plates placed on the bone, into the interstices of which the periosteum gives off prolongations. If the inflammation has its seat in the inner lamella of a tubulated bone, the medullary cavity is narrowed by the exudation given out. A higher degree of inflammation causes a fibrinous product, or a purulent product varying from a thin to a thick fluid, of a yellow or reddish colour, or a product of a greenish, brownish, discoloured and sanious appearance. In such inflammations, which run a very rapid course, the periosteum over the bone seems to be displaced, and frequently distended by pus into a fluctuating sack; in correspondence with the effusion, which is poured out into all parts of its structure, the bone presents an ash-coloured, dirty-yellowish, or reddish-green appearance; in case the effusion is sanious the surface of the bone is rough and corroded.

4. *Caries.*—The sanious bone examined in the recent state presents various appearances, according to the progress which the disease has made. In superficial caries the compact bone is rough and corroded under a covering of sanies; its medullary canals are unequally dilated; the tissues contained in them are partly reduced to a mere friable mass, or flabby warty excrescences, which readily bleed, are developed from them, which shoot outwards in considerable quantity over the rough surface of the bone. The bone always appears porous or spongy, according to the contents of the medullary canals, in the first case discoloured, in the second case rough in various ways. In case of caries of the spongy texture, the bone, when the formation of granulation is luxuriant, assumes a dark, livid redness, it becomes soft, and resembles a piece of flesh permeated by a fine, delicate, bony texture. According to Delpech, Berard, Pouget and Sanson, and according to Mouret, a peculiar fatty substance is produced, in which case, however, according to the last observer, the gelatine has not disappeared from the bone. The carious bone in the macerated and dried state appears rough, as if corroded, and acquires a spongy, porous, worm-eaten appearance, by reason of the unequally-dilated medullary canals, which in several places have formed into holes, and which perforate it; the cells of its spongy substance are dilated, their parietes are attenuated and broken down like the grating of trellis-work.

5. *Osteoporosis.*—In consequence of an excessive development of the marrow or of the tissues which fill the medullary canals and cells of the bone, the bone assumes an increase of volume, its tissue being rarefied. The parietes of the dilated interstices of the bone are so very much attenuated, that gaps or chasms take place at length in the interior and in the external covering, whereby the cavities in the bone enter into communication with each other; the enlarged bone, the higher the degree of the disease is, becomes so much softer, porous and spongy, that it yields to the pressure of the finger, and is easily cut with a knife; its spaces become filled with a dark red medulla collected in great quantity, which is traversed by dilated vessels.

In softening of the bone, which occurs under the two forms of rickets and osteomalacia, we have present an osteoporosis in a different degree; at the same time, however, we have, as an essential anomaly, a reduction of the bone to its cartilaginous element with or without a disturbance in the chemical composition of the same; the bones are accordingly not brittle, but easily bent, they are subject to curvatures, and they are liable not to what may strictly be called fractures, but with more propriety cracks.

6. In case of rickets the bones appear swollen, and the angular shaft of the long bones becomes round, cylindrical; the articulations of the same, and the broad bones, as the pelvic bones, become unusually thick. With respect to the texture of rickety bones, they are affected in two ways, 1st. with osteoporosis accompanied with increase of volume, in which case a pale yellow, reddish jelly is effused into the dilated canals and cells; the vascular bone appears dark-coloured, and somewhat red; this state sometimes attains such a degree, that the cells of the spongy bones and those in the interior of the medullary tubes run together into a larger cavity in consequence of the excessive distension of their parietes, and entirely disappear. 2. The bone sometimes becomes so poor in the amount of the fixed salts, that it becomes entirely reduced to its cartilaginous element, and comes to resemble a bone treated with acids; the bony corpuscles are empty; the lamellar structure is obliterated in some places, and in other places the lamellæ have, as it were, receded from each other, and the bony corpuscles between them are as it were enveloped by them.

7. In osteo-malacia the bones diminish in size and the change of structure consists in osteoporosis with atrophy, a soaking of the bone in fat, and in a reduction of the bone to its cartilaginous element. In this cartilage the bony corpuscles appear empty, the lamellar structure has disappeared ; at the same time the cartilage undergoes a peculiar change in its chemical composition, as the extract obtained by boiling is distinct both from chondrin and from gelatine of bone.

8. In *consecutive sclerosis*, the anomaly of the chemical composition appears to consist in a supersaturation of the cartilaginous element of the sclerotic bone with mineral principles, in the presence of unusual salts, and in an alteration in the habitude of the jelatine of the bone itself. Those cases of sclerosis are particularly marked, as Rokitansky observes, which proceed from osteoporosis occurring in advanced life, and which may oftentimes be observed in the skull. The bones present externally, especially on the cut surface, a chalky appearance with a dull white colour, and on the broken surface a coarse granular appearance ; the bone exhibits irregularly angular medullary canals, the lamellar structure being deficient or perceptible only here and there, the bone-corpuscles lying irregularly one amongst the other. Sclerosis of the head of the thigh-bone in the disease malum coxæ senile exhibits a similar appearance ; the mass of bone takes on a gypsum-like polish. A closer examination detects a thick lamellated structure, the lamellæ very numerous, few bone-corpuscles, which, however, in some places, lie together in thick heaps. The stalactite chalky osteophytes, found on the head of the thigh-bone, exhibit a similar structure, and very many thick, black bone corpuscles, mostly of a rounded form. The sclerosis, connected with rachitism, is characterised by hardness, by a vitreous brittleness of the bone, by a splintery fracture, the medullary canals are small, surrounded by a large, comprehensive, lamellated system, and only a few bone-corpuscles have been observed surrounding the canal ; they are small, and, what is still more remarkable, they are in a great measure transparent.

*Morbid Structures.*

1. *Formation of Cysts.*—The simple cyst, with serous or synovial contents, chiefly in the bones of the face ; *the compound Cystoids ; the Acephalocysts* ; these, like the preceding, are rare phenomena ; Acephalocysts have been seen in the humerus, tibia, os ilium, and in the diploë of the thigh-bone. Rokitansky observes of the specimen preserved in the Vienna collection, that the left os ilium was changed into a serous sack as large as a man's fist, which was filled with numerous pieces of bone of different sizes adhering to the inner wall of the sack, and with echinococus cysts, some being the size of a millet-seed, some that of a nut.

2. *Fibrous Tumours.*—These sometimes attain a very considerable size, distend the bone to a cyst, or crush it in such a manner that dismembered fragments of it are found in its substance, separated the one from the other ; the structure of the fibroid is sometimes thick, sometimes loose, white and elastic.

3. *Enchondroma.*—This is more frequently observed in the bones than in other structures, more especially in the bones of the fingers, toes, ribs, and sternum. It continues like the permanent cartilages for a long time, even during life, in its original state, sometimes it becomes ossified, sometimes it is attacked with inflammation from the surrounding soft parts, and suppurates.

4. *Osteoid.*—This is characterised as a bone developing itself from an ossifying cartilaginous element of new structure in the old bone, in the form of a round tumour, which is distinguished from the normal bony structure by a different elementary texture. Lastly, among the morbid formations have been classed the very rare phenomenon of choleostoma, tubercle, sarcoma, and carcinoma.

---

The enquiries made by several chemists into the nature of diseased bones should now be set down in the same order in which the physical changes of structure of these same bones have been described by Rokitansky.

1. *Exostosis.*—According to Valentine and Lassaigne the quantity of phosphate of lime contained in an exostosis is diminished, whilst the quantity of carbonate is considerably increased; the amount of organic matter is greater than in the bones to which the exostosis is attached. Lassaigne found the following proportions in the bone and in the exostosis.

| | In the bone. | In the exostosis. |
|---|---|---|
| Organic Substance | 41,6 | 46 |
| Phosphate of Lime | 41,6 | 30 |
| Carbonate of Lime | 8,2 | 14 |
| Soluble salts | 8,4 | 10 |

2. On the subject of carious bones Valentine has instituted some experiments, of which the following are the results:

| | 1. | 2. | 3. | 4. |
|---|---|---|---|---|
| Organic Constituents | 55,880 | 54,390 | 45,620 | 54,830 |
| Basic phosphate of Lime | 34,383 | 39,393 | 45,451 | 33,914 |
| Carbonate of Lime | 6,636 | 4,620 | 5,683 | 7,602 |
| Phosphate of Magnesia | 1,182 | 0,520 | 1,180 | 0,389 |
| Chloride of Sodium | 1,919 | 0,424 | 1,620 | 3,157 |
| Carbonate of Soda | | 0,647 | 0,446 | 0,118 |

The first analysis is that of the tibia of a man, 38 years of age; the second is that of the carious external condyle of the femur of a young girl; the third, that of the carious head of the tibia of the same girl; the fourth, that of the carious dorsal vertebra of a man 20 years of age. In all these analyses the organic constituents appear increased. The phosphate of lime is most diminished, the carbonate of lime inconsiderably so. In an osteophyte-like crust deposited around a carious tibia, Valentine found the phosphate of lime still more diminished, and a considerable quantity of chloride of sodium.

We omit any notice of an analysis of *osteoporosis* of the skull, as presenting nothing very remarkable.

4. *Rachitis.*—The bones in rachitis have been frequently examined. They are chiefly characterised by the small quantity of fixed salts they contain. Davy found the following proportions of earthy salts in rickety bones:—

| | |
|---|---|
| In the femur . . . . . . . | 37,8 |
| In the spinous processes . . | 40,7 |
| In the ribs . . . . . . . . | 40,8 |
| In the tibia . . . . . . . | 26,0 |
| In the parietal bone . . . . | 27,1 |

Lehmann (Physiologisch. Chemie. Bd. 1, p. 111.) found in the tibia of a rickety child 33,9 of phosphate of lime.

Marchand (Journal f. Pract. Chemie. October, 1842,) examined several bones of a child who died of rickets; the result of these enquiries was, that the spinal vertebra lost most, the sternum least of the fixed salts.

Marchand found the cartilage of the rickety bone so essentially changed, that on being boiled it yielded a fluid containing neither gluten nor chondrine.

Marchand, on examining the urine of the child, whose bones were subjected to experiment by him, as we have just now seen, found a short time before death five or six times more earthy phosphates than are found in healthy urine. Berzelius shewed how extremely easily the phosphate of lime is dissolved by lactic acid. As in scrofula and in scrofulous bones an excessive formation of acid is generated in the primæ viæ, it is not improbable that a greater quantity of acid reaches the circulation, and the proximate cause of the solution of the bone-earth emanates from the bones. So long as the alkaline salts of the blood are sufficient to satiate the excess of acid, so long are the bones preserved in their healthy state; but if this is not the case, their bone-earth becomes dissolved. Marchand very justly animadverts on the absurd treatment of rickety children with madder. The natural mode of treatment should strive to meet the cause of the excessive generation of acid by suitable diet and antacids, and in the next place all those alimentary substances which are capable of forming lactic acid, as sugar, starch, and gum, must be avoided.

In *osteomalacia,* as in rickets, the earthy salts in the bones are very much diminished.

Ragsky has made several experiments with *sclerotic* bones. In simple sclerosis of the skull in a madman the sp. grav. of the bone was 1,911. On boiling, the cartilage was converted slowly into jelly, the solution was whitish, turbid, and gelatinous; alcohol produced great turbidness in it, and tincture of galls a considerable precipitate.

We now shall pass on to the consideration of *Arthritic Bones.* It is well known, that the deposits which form in consequence of arthritic disease on the bones, and chiefly on the ends of the joints, are combinations of uric acid, and also that the diseased bones themselves contain a less quantity of fixed salts than healthy bones. Marchand examined the thigh-bone and the fore-arm of an arthritic individual: the analysis was as follows:—

| | Thigh-bones. | Bones of the Fore-arm. |
|---|---|---|
| Animal substance | 46,32 | 45,96 |
| Phosphate of lime | 42,12 | 43,18 |
| Carbonate of lime | 8,24 | 8,50 |
| Phosphate of magnesia | 1,01 | 0,99 |
| Fluoride of calcium, Soda, chloride of sodium, Loss | 2,31 | 1,37 |
| | 100,00 | 100,00 |

The thigh-bone was covered with deposits at the end of the joint, the bones of the fore-arm, on the contrary, were in the normal state; still both bones evince a corresponding deviation from the normal composition, so that the morbid process is expressed not merely in the bones on which deposits exist, but over the entire bones of the body; the similar diminution of the fixed salts, and chiefly of the earthy phosphates, is obvious here. Exostoses of arthritic bones are occasioned not by a growing out of the bony substance, but by the expulsion of matters foreign to the normal composition of the bone. The concretions found on the arthritic thigh-bones above noticed, were examined by Marchand; who found therein:—

| | |
|---|---|
| Urate of soda | 34,20 |
| Urate of lime | 2,12 |
| Carbonate of ammonia | 7,86 |
| Chloride of sodium | 14,12 |
| Water | 6,80 |
| Animal substance | 32,53 |
| Loss | 2,37 |
| | 100,00 |

Ragsky examined a gypsum-like concretion on the head of the thigh-bone of a person labouring under malum coxæ senile; its sp. grav. was 0,845; on boiling, it yielded a brownish-yellow transparent fluid, which evinced but little disposition to gelatinise, but from which a large precipitate was thrown down by tincture of galls and chloride of platinum; it contained—

| | |
|---|---|
| Organic constituents | 33,90 |
| Basic phosphate of lime, with phosphate of magnesia | 59,10 |
| Carbonate of lime | 6,57 |
| Salts soluble in water | 0,43 |

No uric acid could be found in it.

An interesting case of the disease of bone, to which Joh. Müller has given the name of *Osteoid*, is here communicated by the author. It occurred in a boy 14 years of age, of a very leucophlegmatic appearance; he was labouring at the time of his admission under dropsy of various parts of the body; there was formed around the knee-joint a knobby hard swelling. On close examination it was found that the tibia, patella, &c., were entirely free of disease, and that the tumor was seated on the os femoris, on which it extended almost from the condyles to the head of the joint. The limb was amputated, and the structure of the tumour was minutely examined; on being sawn through, it was found to consist of two strata. The first, which had its seat immediately on the diseased bone itself, presented a hard mass, similar to compact bony substance, only in some parts it was permeated by a somewhat softer mass; this mass immediately beneath the periosteum raised itself in the form of straight bony fibres, and passed gradually into the substance of the bone; the second stratum or layer, composing this tumour, and forming its external part, was softer, and of a pulpy consistence, still, however, resisting pressure; it appeared to be entirely similar in structure to the hard layer beneath it, and to differ from it only in containing a smaller quantity of bone earth. On submitting the several parts of this tumour to chemical analysis, after first removing the fat, and performing the other preliminary steps, the results obtained mainly consisted in this; the greatest difference in the composition of this morbid formation consisted in the amount of the carbonate of lime it contained, which appears to be always the first constituent diminished in diseases of the bones; the salts soluble in water are increased, and indeed so much the more, the less the quantity is of the salts of lime in the morbid product. A more detailed account of the chemical changes observed in this case would appear uninteresting to the majority of our readers.

